# Efficacy and safety of monoclonal antibody therapy in patients with neuromyelitis optica spectrum disorder: A systematic review and network meta-analysis

**DOI:** 10.3389/fneur.2023.1166490

**Published:** 2023-04-04

**Authors:** Saharat Aungsumart, Sitaporn Youngkong, Charungthai Dejthevaporn, Usa Chaikledkaew, Kunlawat Thadanipon, Amarit Tansawet, Jedsada Khieukhajee, John Attia, Gareth J. McKay, Ammarin Thakkinstian

**Affiliations:** ^1^Mahidol University Health Technology Assessment (MUHTA) Graduate Program, Mahidol University, Bangkok, Thailand; ^2^Department of Neurology, Prasat Neurological Institute, Bangkok, Thailand; ^3^Social and Administrative Pharmacy Division, Department of Pharmacy, Faculty of Pharmacy, Mahidol University, Bangkok, Thailand; ^4^Division of Neurology, Department of Medicine, Faculty of Medicine, Ramathibodi Hospital, Mahidol University, Bangkok, Thailand; ^5^Department of Clinical Epidemiology and Biostatistics, Faculty of Medicine, Ramathibodi Hospital, Mahidol University, Bangkok, Thailand; ^6^Department of Surgery, Faculty of Medicine Vajira Hospital, Navamindradhiraj University, Bangkok, Thailand; ^7^School of Medicine and Public Health, Faculty of Health and Medicine, University of Newcastle, Callaghan, NSW, Australia; ^8^Centre for Public Health, School of Medicine, Dentistry and Biomedical Sciences, Queen's University, Belfast, United Kingdom

**Keywords:** neuromyelitis optica spectrum disorder (NMOSD), FDA-approved monoclonal antibodies (mAbs), off-label monoclonal antibodies, relapse, network-meta analysis

## Abstract

**Introduction:**

Neuromyelitis optica spectrum disorder (NMOSD) is a devastating inflammatory CNS demyelinating disease. Two groups of monoclonal antibodies (mAbs) are used to prevent disease relapse, i.e., Food and Drug Administration (FDA)-approved mAbs (e.g., eculizumab satralizumab, inebilizumab), and off-label mAb drugs (e.g., rituximab and tocilizumab). The FDA-approved mAbs have high efficacy but more expensive compared to the off-labels, and thus are less accessible. This systematic review and network meta-analysis (NMA) was to assess the efficacy and safety of both classes of mAbs compared to the current standard treatments.

**Methods:**

Systematically searches were conducted in MEDLINE and SCOPUS from inception until July 2021. Randomized-controlled trials (RCTs) were eligible if they compared any pair of treatments (mAbs, immunosuppressive drugs, or placebo) in adult patients with NMOSD. Studies with AQP4-IgG positive or negative were used in the analysis. Probability of relapse and time to event were extracted from the Kaplan-Meier curves using Digitizer. These data were then converted into individual patient time-to-event data. A one-stage mixed-effect survival model was applied to estimate the median time to relapse and relative treatment effects using hazard ratios (HR). Two-stage NMA was used to determine post-treatment annualized relapse rate (ARR), expanded disability status score (EDSS) change, and serious adverse events (SAE). Risk of bias was assessed using the revised cochrane risk of bias tool.

**Results:**

A total of 7 RCTs with 776 patients were eligible in the NMA. Five of the seven studies were rated low risk of bias. Both FDA-approved and off-label mAbs showed significantly lower risk of relapse than standard treatments, with HR (95% CI) of 0.13 (0.07, 0.24) and 0.16 (0.07, 0.37) respectively. In addition, the FDA-approved mAbs had 20% lower risk of relapse than the off-label mAbs, but this did not reach statistical significance. The ARRs were also lower in FDA-approved and off-label mAbs than the standard treatments with the mean-difference of−0.27 (-0.37,−0.16) and−0.31(-0.46,−0.16), respectively.

**Conclusion:**

The off-label mAbs may be used as the first-line treatment for improving clinical outcomes including disease relapse, ARR, and SAEs for NMOSD in countries where resources and accessibility of the FDA-approved mAbs are limited.

**Systematic review registration:**

https://www.crd.york.ac.uk/prospero/display_record.php?RecordID=283424, identifier: CRD42021283424.

## Introduction

Neuromyelitis optica spectrum disorder (NMOSD) is a devastating central nervous system (CNS) inflammatory demyelinating disease that approximately seventy-five percent is caused by autoantibodies targeting aquaporin-4 immunoglobulin G (AQP4-IgG) (1). Patients usually present with severe optic neuritis and myelitis, which can cause blindness and quadriplegia (2). The extent of the disability depends on the frequency and severity of disease relapses. Thus, immunosuppressive and immunomodulatory drugs are essential treatments for relapse prevention in patients with NMOSD.

Standard immunosuppressive medication considered initial treatments for relapse prevention especially poor resource countries include prednisolone, azathioprine, and mycophenolate mofetil. However, many patients still relapse because of low efficacy and low adherence related to severe side effects (3). Monoclonal antibodies [mAbs: i.e., eculizumab, (4) satralizumab (5, 6), inebilizumab (7)] have more recently been approved by the Food and Drug Administration (FDA) for NMOSD, and are claimed to have higher efficacy and lower side effects, but at much higher cost than the standard immunosuppressive drugs, making them less accessible. Therefore, other mAbs [i.e., rituximab (8, 9) and tocilizumab (10)] are used as “off-label” medications because of their much lower cost than the approved mAbs.

Fifteen systematic reviews (SRs) have evaluated treatment effects of mAbs on relapse prevention in NMOSD (11–25). However, most SRs were performed using data from observational studies; only a single direct meta-analysis (19) included only randomized control trials (RCTs). Two SRs also applied network meta-analysis (NMA) (13, 23); one published in 2019 had a mix of one RCT and five observational studies to compare the efficacy of four immunosuppressive drugs (i.e., cyclosporine, azathioprine, mycophenolate mofetil, and cyclophosphamide) with a single mAb (i.e., rituximab) (13). Another NMA was recently published considering only four FDA-approved mAbs (23). Several RCTs that assessed the effects of off-label mAbs were published after this most recent NMA. To our knowledge, there is no evidence comparing efficacy and safety of all off-label mAbs with all FDA-approved drugs and standard immunosuppressive drugs. Therefore, this SR-NMA was conducted to compare the treatment efficacy (i.e., relapse and disability progression) and serious adverse events (SAE) among FDA-approved and off-label mAbs, and also among immunosuppressive drugs.

## Methods

A review protocol of this SR-NMA was developed and registered with PROSPERO (CRD42021283424). The study was conducted following the Preferred Reporting Item for Systematic reviews and Meta-Analysis (PRISMA) guidelines (26). Relevant studies were independently identified by two reviewers (SA and KT) from MEDLINE and SCOPUS databases from inception to 30th July 2021. The search terms were constructed based on patients, interventions, outcomes, see Supplementary Tables 1.1, 1.2.

RCTs were selected if they met all of the following inclusion criteria: (i) RCTs included adults with NMOSD; (ii) compared any pair of mAbs (rituximab, inebilizumab, tocilizumab, satralizumab, or eculizumab), immunosuppressive drugs (azathioprine, mycophenolate mofetil, cyclophosphamide, cyclosporine, etc.), or placebo; (iii) had at least one of the following outcomes: time to relapse, annualized relapse rate (ARR), expanded status disability scale (EDSS) change after treatment, and SAE. Studies were excluded if published in a foreign language that could not be translated, compared dosages of the same drug, or had insufficient data for pooling after three contact attempts with authors.

### Interventions

Interventions of interest were any off-label mAbs (rituximab, tocilizumab) and approved mAbs (inebilizumab, satralizumab, or eculizumab), whereas the control treatments could be any of the recognized standard treatments (i.e., cyclosporine, azathioprine, mycophenolate mofetil, and cyclophosphamide), placebo, or no treatment. For those RCTs with a placebo arm plus standard treatments, their comparator was categorized as the standard treatment.

### Outcomes

The primary outcome of interest was time to relapse, defined as the time since randomization to disease relapse. Relapse was defined according to the individual studies, such as worsening or appearance of new neurological symptoms 24 h or more post intervention, without evidence of fever or infection (27). The adjudicated relapse was consider as primary analysis instead of clinician reported relapse. The secondary outcomes were post-treatment ARR, EDSS changed from baseline, and SAEs. ARR was defined as the number of relapses within 1 year. The EDSS, a neurological disability scale, measured by clinicians to assess NMOSD progression (28), ranges from 0.0 (normal neurological status) to 10.0 (death). SAEs were defined as death or any life-threatening condition, required or prolonged hospitalization, persistence or significant disability, or congenital anomaly.

### Data extraction and quality assessment

Two independent reviewers performed the data extraction and quality assessment (SA and JK). The following data were extracted: general characteristics (i.e., number of patients, type of study, RCT phase, country), patient characteristics (i.e., age, age at onset, gender, disease duration, the previous annualized relapse rate, baseline EDSS score, and AQP4 positive), treatment regimens, and outcomes (i.e., time to first relapse, post-treatment ARR, EDSS change, and SAE).

Data for time to relapse were extracted from the Kaplan-Meier (KM) curve using the Digitizer program (29) including probabilities, times to first relapse, number of events, and person-time at risk at each distinct time. These data were then used to generate an individual's time to event data point (30). Quality control was applied to check if the generated raw data were valid including: number of converted events (i.e., relapse) should not exceed the original number reported in the article, and summary statistics (e.g., relapse rate, hazard ratio [HR]) estimated from the generated data should be close to those reported in the original article. The risk of bias was assessed using the Revised Cochrane Risk of Bias tool (RoB2) to assess the quality of RCTs. Any disagreement in data extractions or RoB was resolved by consensus with a third reviewer (ATh).

### Statistical analysis

A one-stage mixed-effect parametric survival model (31) was applied to pool relative treatment effects across studies. Various distributions of relapse time were applied to estimate time to relapse including Weibull, exponential, log-normal, gamma, and log-logit distributions. Akaike's Information Criteria (AIC) were applied to select the best performing distribution. The relative treatment effects (i.e., HR and median relapse time) were estimated using Weibull survival regression.

For secondary outcomes (i.e., ARR, change of EDSS, and SAE), the relative treatment effects were estimated for continuous and dichotomous outcomes by unstandardized mean difference (USMD) and risk ratio (RR), respectively. A two-stage NMA with a consistency model was applied to pool these relative treatment effects across treatment regimens and studies. The probability of being the best treatment was assessed using the rankogram and the Surface Under the Cumulative Ranking curve (SUCRA). Transitivity was explored by comparing the distribution of co-variables (e.g., gender, age, duration, AQP-4 status, previous ARR, and diagnosis with neuromyelitis optica) among treatment arms and studies. The inconsistency was checked by the design-by-treatment interaction model. A comparison-adjusted funnel plot was used to assess publication bias. All analyses were performed using STATA version 17.0. A two-sided *p*-value < 0.05 was considered statistically significant for all tests, except for heterogeneity where 0.10 was used.

## Result

### Studies characteristics

**Figure 1:**
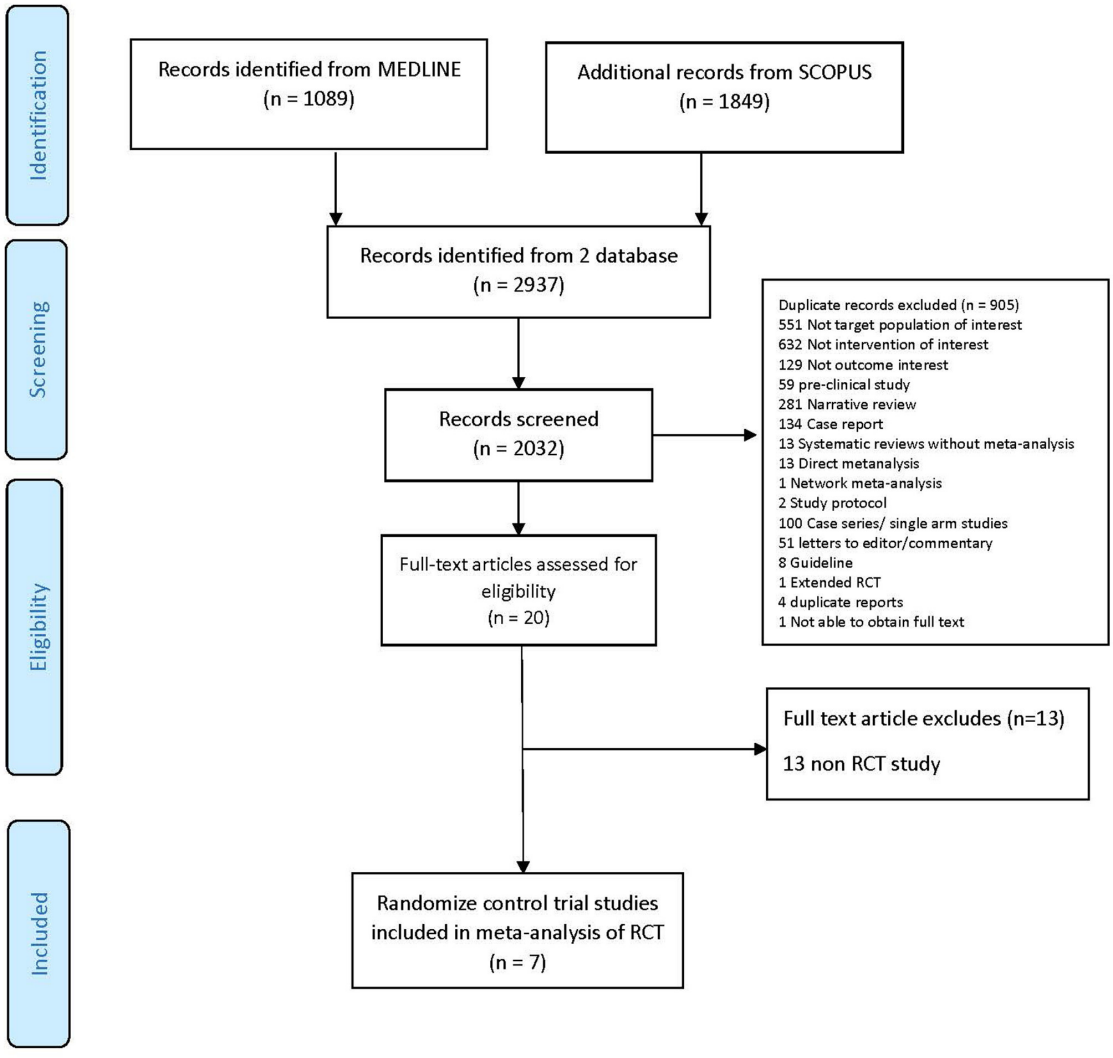
PRISMA flow diagram.

A total of 2,937 studies were identified but only 7 RCTs (4–10) with 776 patients were eligible for inclusion; reasons for exclusion are reported in the PRISMA flow diagram (Figure 1). The characteristics of the RCTs included are presented in Table 1. Mean age ranged from 32 to 45 years, and most participants were female (73 to 100%). Mean previous ARR ranged from 1.0 to 2.1, and percentage AQP4-IgG positive varied from 39 to 100%. Only 3 RCTs reported phenotype of patients whether NMO or NMOSD, which ranged from 72% to 91%.

**Table 1:** Study characteristics.

**Authors**	**Interventions**		**RCT phase**	**Follow-up time (weeks)**	***n***	**Country/ region**	**% female**		**Mean age**		**Previous ARR**		**% AQP4-IgG positive**		**% NMO**		**Outcomes**
	**mAb (dose)**	**Standard immuno-suppressant**					**mAb**	**Control**	**mAb**	**Control**	**mAb**	**Control**	**mAb**	**Control**	**mAb**	**Control**	
Nikoo et al. (8)	Rituximab (1,000 mg IV every 2 weeks, then every 6 months)	AZA	2	52	68	Iran	87.9	80	35.3	32.4	1.3	1.0	39	57	-	-	pARR, cEDDS, SAE
Pittock et al. (4)	Eculizumab (900 mg IV weekly, then 1,200 mg every 2 weeks)	Any standard immune-suppressants	3	90.9	143	18 countries US, Eur, AP	92	89	43.9	45.0	1.9	2.1	100	100	72	91	Relapse, pARR, cEDDS, SAE
Cree et al. (7)	Inebilizumab (300 mg IV at day 1 and day 14)	None	2/3	28.1	230	25 countries US, Eur, AP, Aus/NZ	91	89	43.0	42.6	1.7	1.6	92	93	80	67	Relapse, SAE
Yamamura et al. (5)	Satralizumab (120 mg SC at week 0, 2, 4, and every 4 weeks)	Any standard immune-suppressants	3	107.4	83	11 countries US, Eur, AP	90	95	40.8	43.4	1.5	1.4	66	67	-	-	Relapse, pARR, cEDDS, SAE
Tahara et al. (9)	Rituximab (375 mg/m^2^ IV weekly for 4 weeks, then 1,000 mg every 6 months)	None	2	72	38	Japan	90	100	51.5	49	1.7	1.1	100	100	-	-	Relapse, pARR, cEDDS, SAE
Traboulsee et al. (6)	Satralizumab (120 mg SC at week 0, 2, 4, and every 4 weeks)	None	3	92.3	168	13 countries US, UK, AP	73	97	45.3	40.5	1.4	1.5	65	72	75	75	Relapse, pARR, cEDDS, SAE
Zhang et al. (10)	Tocilizumab (8 mg/kg IV every 4 weeks)	AZA	2	60	118	China	93	90	48.1	45.3	1.7	1.7	85	90	-	-	Relapse, SAE

AP, Asia-Pacific; AQP4-IgG, Aquaporin4-IgG; ARR, Annualized relapse rate; Aus/NZ, Australia and New Zealand; AZA, Azathioprine; cEDSS, expanded disability status score change; Eur, Europe; IV, intravenous; mAb, monoclonal antibody; NMO, neuromyelitis optica; pARR, post-treatment Annualized relapse rate; SC, subcutaneous; RCT, Randomized-controlled trial; US, United States.

The risk of bias for each study is shown in the Supplementary Figure S1 (32). Most studies (5/7) had low risk of bias. All studies had low risk from the randomization process, while two (8, 10) and one (8) were high risk due to deviation from intended interventions, missing outcome data, or some concern with outcome measurement.

### Time to first relapse

**Figure 2:**
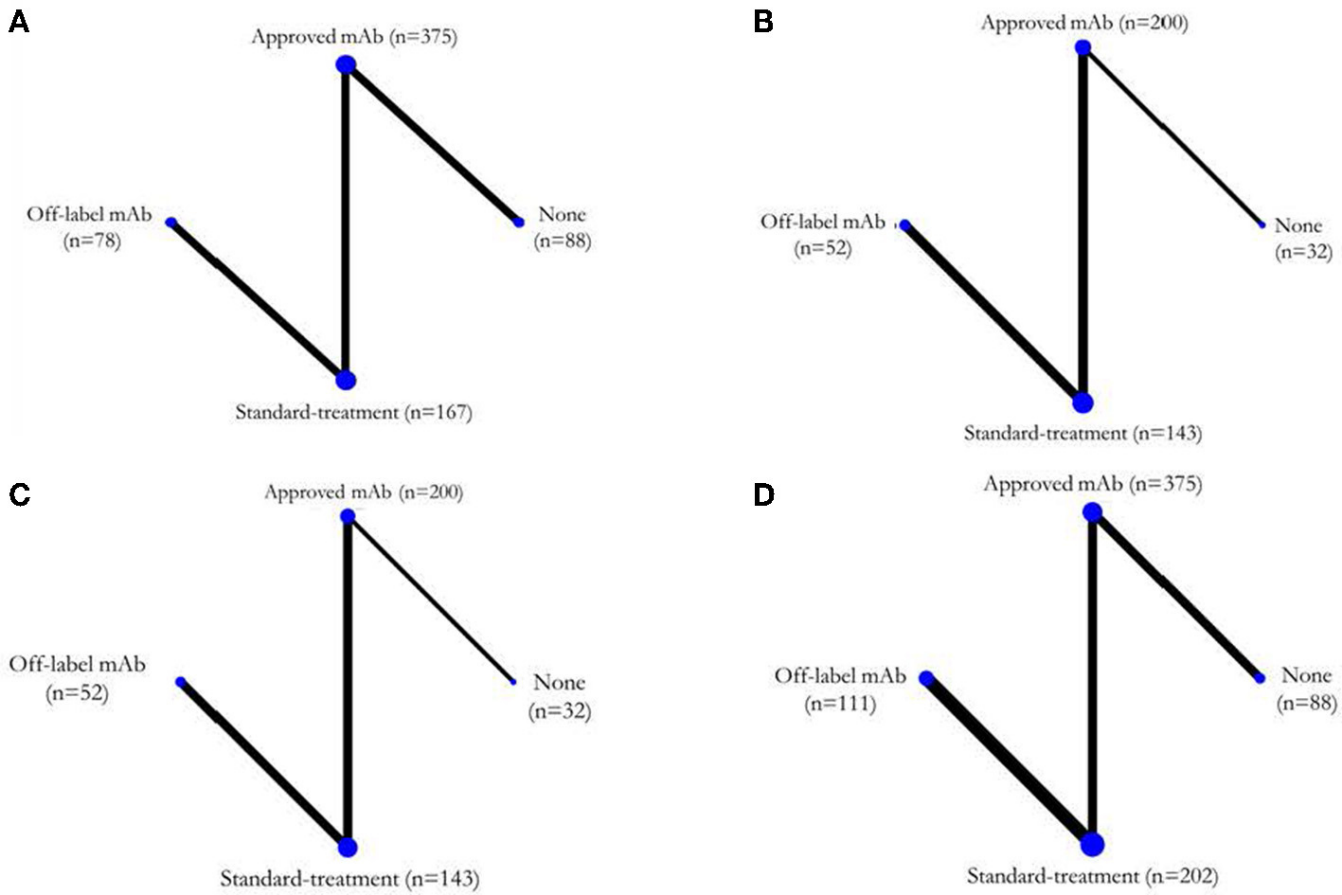
Network map for each outcome. **(A)** Time to disease relapse, **(B)** ARR post-treatment, **(C)** EDSS changes, and **(D)** SAEs. ARR, annualized relapse rate; EDSS, expanded disability status score; SAEs, serious adverse events.

**Figure 3:**
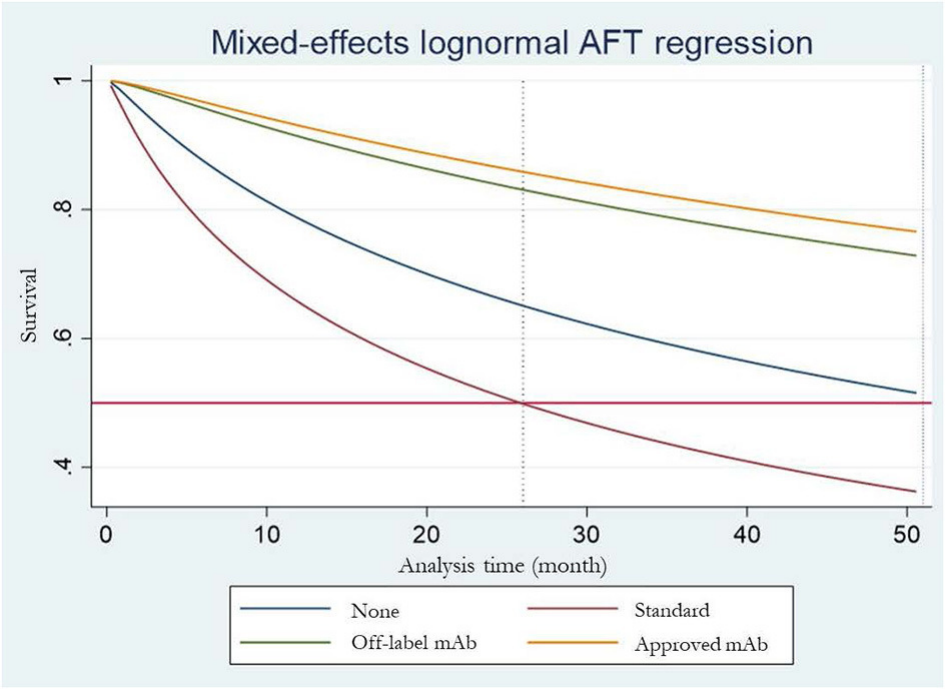
Estimated median time to first release for each treatment arm.

Six (4–7, 9, 10) of the seven RCTs reported KM curves of time to relapse by treatment group. Individual patient time to relapse data for 707 patients were generated, which consisted of 4 treatment arms including no treatment, standard treatment (prednisolone or any immunosuppressive drugs), off-label mAbs, and approved mAbs, see Figure 2A. The one-stage NMA with a mixed-effect accelerated failure time model was applied with various distributions; a log-normal distribution was the best (i.e., lowest AIC), see Supplementary Table S3. A relapse-free survival probability curve was constructed by treatment group; this indicated highest relapse-free survival with approved mAbs, followed closely by off-label mAbs, with no-treatment being the worst (Figure 3). Predicted median times to relapse were 25.8 (11.6,40.1) and 54.4 (15.4, 93.4) months for the standard treatment and no-treatment group, compared to longer than 55 months in both approved and off-label mAbs. Relative treatment effects for regimens were estimated (Table 2) indicating patients receiving approved and off-label mAbs had 0.27 (0.15, 0.48) and 0.34 (0.11, 0.99)-fold significantly lower relapse than the no-treatment group. Conversely, patients who received the standard treatment were at 2.11 (0.96, 4.63) fold higher risk of relapse than no-treatment but this was not significant. In addition, the approved and off-label mAbs also had lower risk of relapse, i.e., 0.13 (0.07, 0.24) and 0.16 (0.07, 0.37)-fold than the standard treatments. The approved mAbs had slightly lower risk of relapse relative to off-label mAbs (0.80 [0.31, 2.07]), but this did not reach statistical significance. The SUCRA indicated the best treatment in lowering relapse was approved-mAbs, followed by off-label mAbs, with SUCRA of 85.6 and 68.2, respectively.

**Table 2:** Hazard ratio and 95% confidence interval of network meta-analysis of time to clinical outcomes (above gray diagonal line)^a^.

**Time to relapse**			
No-treatment 37.8 (3%)	2.11 (0.96, 4.63)	0.34 (0.11, 0.99)^b^	0.27 (0.15, 0.48)^b^
	Standard-treatment 8.5 (0%)	0.16 (0.07, 0.37)^b^	0.13 (0.07, 0.24)^b^
		Off-label mAbs 68.2 (38%)	0.80 (0.31, 2.07)
			Approved mAbs 85.4 (59%)
**Post treatment ARR**			
No-treatment	0.03 (−0.18, 0.24)	−0.28 (−0.54, −0.03)^b^	−0.24 (−0.42, −0.06)^b^
	Standard-treatment	−0.31 (−0.46, −0.16)^b^	−0.27 (−0.37, −0.16)^b^
		Off-label mAbs	0.04 (−0.14, 0.23)
			Approved mAbs
**EDSS change**			
No-treatment	0.10 (−0.44, 0.65)	0.14 (−0.46, 0.73)	−0.17 (−0.62, 0.28)
	Standard-treatment	0.03 (−0.21, 0.28)	−0.27 (−0.58, 0.03)
		Off-label mAb	−0.31 (−0.70, 0.09)
			Approved mAb
**SAEs**			
No-treatment	0.93 (0.39, 2.20)	0.59 (0.19, 1.79)	0.83 (0.41, 1.70)
	Standard-treatment	0.64 (0.32, 1.28)	0.90 (0.55, 1.45)
		Off-label mAb	1.41 (0.60, 3.30)
			Approved mAb

ARR, annualized relapse rate; EDSS, expanded disability status score; mAbs, monoclonal antibodies; SAEs, serious adverse events.

a Results of treatment comparisons are read from right to left. Value in the gray diagonal line are SUCRAs, whereas the probability of being the best treatment in lowering relapse is shown in parenthesis.

b Statistical significance, *p* = 0.05.

The comparison-adjusted funnel plot for time to relapse is shown in the Supplementary Figure S2A. The study of Tahara (9) was excluded from this plot because it was not possible to plot the very small standard error in the graph. The result suggested no small-study effect.

### Post-treatment ARR

Post-treatment ARR assessment was based on five studies (4–6, 8, 9) (*n* = 427) (Figure 2B). The USMD of post-treatment ARR was estimated with approved-mAbs representing a post-treatment ARR of −0.24 (−0.42, −0.06) and −0.27 (−0.37, −0.16), significantly lower than no-treatment and standard treatments, respectively (see Table 2). Likewise, the off-label mAb ARR was −0.28 (−0.54, −0.03) and −0.31(−0.46, −0.16) vs. the same comparators. However, mean post-treatment ARRs between approved-mAbs and off-label mAbs were not significantly different with USMD of 0.04 (−0.14, 0.23). In addition, the comparison-adjusted funnel plot provided no evidence of asymmetry and evidence of publication bias (Supplementary Figure S2B).

### EDSS change

EDSS change assessment was based on five studies (4–6, 8, 9) (*n* = 427) (Figure 2C). The USMD of EDSS change was estimated, indicating approved-mAbs had EDSS change of −0.17 (−0.62, 0.28) and −0.27 (−0.62, 0.28) lower than no-treatment and standard treatments, respectively (Table 2). In contrast, off-label mAb had a 0.14 (**-**0.46, 0.73) and 0.03 (**-**0.21, 0.28) higher EDSS change than these corresponding comparators. However, none of these comparisons reached statistical significance. A comparison-adjusted funnel plot suggested no evidence of publication bias (Supplementary Figure S2C).

### SAEs

Seven RCTs (4–10) were included in the SAEs analysis (Figure 2D). Most SAEs were required hospitalizations due to severe infections such as pneumonia or sepsis. The SAEs were 0.83 (0.41, 1.70) and 0.90 (0.55, 1.45)-fold lower in approved mAbs than in no-treatment and standard treatment; likewise, off-label mAb were 0.59 (0.19, 1.79) and 0.64 (0.332, 1.28)-fold lower than these corresponding comparators; none of these were statistically significant. The comparison-adjusted funnel plot for SAEs (Supplementary Figure S2D) indicated no asymmetry or risk of publication bias.

## Discussion

We conducted the first NMA of efficacy and safety of current treatments in NMOSD using evidence based solely from RCTs. Our findings suggested that the FDA-approved and off-label mAbs represent an 87% and 84% significantly lower risk of relapse than standard treatments. In addition, both corresponding mAbs had between 0.27 and 0.31 significantly lower ARRs than standard treatments, while outcomes for EDSS and SAEs did not significantly differ.

mAbs are classified into two categories in accordance with FDA approved data: off-label and FDA-approved mAbs. The former group previously demonstrated higher efficacy compared to classic immunosuppression and were considered more accessible than the FDA-approved mAb (8–10). However, previous studies have shown that FDA-approved mAbs were very effective in controlling NMOSD disease activity compared to placebo (4–7, 23). A non-statistically significant difference in disease activity was seen in FDA-approved mAbS compare to the off-label mAbs. Furthermore, a non-statistically significant difference was also observed in reduction of SAE's when FDA-approved and off-label mAbs were compared to standard immunosuppressive drugs.

Surprisingly, a non-statistically significant difference was observed in increasing the risk of relapse when standard immunosuppressive drugs was compared to the placebo (HR 2.11; 95CI 0.96, 4.96). The explanation might possibly cause by heterogeneity of each study design. Some of the study include placebo as immunosuppressive resistance patients, which allow patients to continue use previous immunosuppressive drug (4, 5). These patients group was considered as aggressive NMOSD compare with the naïve NMOSD patients, who include as true placebo in other studies (6, 7).

mAbs can also be classified according to their mechanism of action such as C5 complement inhibitor (e.g., eculizumab), anti-B cell or anti-CD19 (e.g., inebilizumab), and anti-IL-6 (e.g., Satralizumab). For off-label mAbs, rituximab is classified as anti-B cell or anti- CD20, while tocilizumab is anti-IL-6. Most FDA approved and off-label mAbs have similar mechanisms (except for eculizumab) and it was therefore unsurprising that their outcomes did not significantly differ.

Our findings were similar to those from a previous NMA (13), which combined data from both RCTs and observational studies but the later data were mainly included (5/6 studies) in pooling treatment effects demonstrating a significant reduction in ARR and EDSS for rituximab (an off-label mAb) compared to azathioprine. Nevertheless, our study, which included only RCTs, provides more robust and better-quality evidence. In addition, we provided additional evidence of time to relapse and risk of relapse based on individual patient data generated from KM curves.

Our findings will inform neurologists and opthalmologists in treatment decision making, perhaps highlighting the possibility for off-label mAb use in preference to FDA-approved mAbs, especially in resource limited settings such as low and low-middle-income countries where accessibility may be limited. FDA-approved mAbs may represent a suitable alternative for patients that suffer from disease relapse following treatment with off-label mAbs.

Our study had several strengths. First, we applied both one- and two-stage NMA approaches to compare FDA-approved mAbs and off-label mAbs indirectly. A total of seven RCTs were included considering all relevant clinical outcomes, including relapse time, ARRs, EDSS, and severe SAEs. New agents (i.e., eculizumab, inebilizumab, satralizumab, and tocilizumab) were also included in the analysis of both FDA-approved and off-label mAbs. Individual patient (time to event) data were generated from the KM curves (30), leading to relative treatment effects as time to relapse and also HR effect estimates. Nevertheless, our study also had several limitations. First, treatments were collapsed under four drug groups rather than individual drugs because of the limited number of RCTs available for inclusion. In particular, comparisons of treatment effects between standard treatments and no-treatment, use of standard immunosuppressive therapies appeared worse than no treatment which may be due to the different effects of individual immunosuppressants, although this was not significant. Second, we could neither assess the optimal dose and nor period of treatment particular in maintenance therapy of off-label mAbs due to limited number of included studies. Finally, our evidence was based on short-term follow-up (ranged from 28.1 to 107.4 weeks), longer follow-up studies are further required to assess long-term effects of off-label mAbs including optimal dose for maintenance, relapse rate, and adverse events such as sino-pulmonary and urinary tract infection.

In conclusion, off-label of the mAbs rituximab and tocilizumab may be used as the first-line treatments for improving clinical outcomes, such as disease relapse, ARR, and SAEs in countries where resources and accessibility to the FDA-approved mAb are limited. The FDA-approved mAbs may be considered as the next line treatments in patients who are failed from off-label mAbs or uncontrolled disease activity. A large-scale RCT of individual mAbs should be further conducted to evaluate optimal-dose, long-term treatment efficacy, and safety with immune suppressants. Furthermore, an economic evaluation should also be conducted to evaluate their clinical and cost-utility for informing treatment decision-making.

## Data availability statement

The raw data supporting the conclusions of this article will be made available by the authors, without undue reservation.

## Author contributions

SA, SY, CD, UC, and ATh conceived and designed the work. SA and KT did the analysis. SA wrote the first draft of the manuscript with input from other authors. All authors interpreted the data, provided critical revision for important intellectual content, and approved the final version to be published.
