# Supplementary material for: Efficacy and safety of monoclonal antibody therapy in patients with neuromyelitis optica spectrum disorder: A systematic review and network meta-analysis
Source: Front Neurol. 2023 Apr 4;14:1166490. doi: 10.3389/fneur.2023.1166490 (PMC10110910; doi:10.3389/fneur.2023.1166490)
Supplement: Supplementary file 1 [file Data_Sheet_1.docx]

**Supplementary appendices**

Efficacy and safety of monoclonal antibody in patients with Neuromyelitis Optica spectrum disorder: A systematic review and network meta-analysis

Saharat Aungsumart, PhD, Sitaporn Youngkong, PhD*, Charungthai Dejthevaporn, PhD, Usa Chaikledkaew, PhD, Kunlawat Thadanipon, MSc, Amarit Tansawet, MD, Jedsada Khieukhajee, MD, John Attia, PhD, Gareth J. McKay, PhD, Ammarin Thakkinstian, PhD

***Correspondance:** Sitaporn Youngkong: sitaporn.you@mahidol.edu

(All figures and tables in the supplementary appendices are created by the authors)

Online Supplementary Content

Contents

[**Appendix 1 Search results** 1](#_Toc81908729)

[**eTable 1.1 MEDLINE database search result**](#_Toc81908732) 1

[**eTable 1.2 SCOPUS database search result**](#_Toc81908733) 3

**[Appendix 2 Risk of Bias assessment](#_Toc81908735)**5

[**eFigure 2.1 Risk of bias asseessment**](#_Toc81908745) 5

**[Appendix 3 Results of Mixed effect parametric survival model using difference distribution](#_Toc81908744)** 6

**[eTable 3.1 Mixed effect parametric survival model using difference distribution](#_Toc81908745)** 6

**[Appendix 4 Funnel plots for each treatment comparison from NMA](#_Toc81908755)**7

[**eFigure 4.1 Funnel plots for each treatment comparison from NMA on time to relapse**](#_Toc81908761) 7

[**eFigure 4.2 Funnel plots for each treatment comparison from NMA on post-treatment annualized relapse rate**](#_Toc81908761) 8

[**eFigure 4.3 Funnel plots for each treatment comparison from NMA on expanded disability status score change** …………………………………………… ……………… 9](#_Toc81908761)

[**eFigure 4.2 Funnel plots for each treatment comparison from NMA on serious adverse events**](#_Toc81908761) ………………………………………………………………………………………..10

**Appendix 5 Individual study data use for analysis**……………………………………...11

**eTable 5.1 Individual study data use for analysis**……………………………………. 11

**Appendix 1 Search results**

**eTable 1.1** MEDLINE database search result

|  | Search number | Query | Results |
| --- | --- | --- | --- |
| P | 1 | neuromyelitis optica | 5,083 |
|  | 2 | "Neuromyelitis Optica"[Mesh] | 3,397 |
|  | 3 | NMO | 2,304 |
|  | 4 | NMOSD | 1,563 |
|  | 5 | Devic's disease | 5,127 |
|  | 6 | #1 OR #2 | 5,083 |
|  | 7 | #1 OR #3 | 5,399 |
|  | 8 | #1 OR #4 | 5,123 |
|  | 9 | #1 OR #5 | 5,127 |
|  | 10 | #1 OR #3 OR #4 | 5,437 |
|  | 11 | #1 OR #3 OR #4 OR #5 | 5,480 |
| I | 12 | azathioprine | 23,726 |
|  | 13 | "Imuran" | 340 |
|  | 14 | #12 OR #13 | 23,800 |
|  | 15 | mycophenolate mofetil | 14,176 |
|  | 16 | MMF | 5,568 |
|  | 17 | "Cellcept" | 207 |
|  | 18 | #15 OR #16 | 15,738 |
|  | 19 | #15 OR #17 | 14,219 |
|  | 20 | #15 OR #16 OR #17 | 15,779 |
|  | 21 | "Myfortic" | 67 |
|  | 22 | #20 OR #21 | 15,786 |
|  | 23 | cyclosporine | 60,084 |
|  | 24 | "Sandimmun*" | 662 |
|  | 25 | "Neoral" | 1,279 |
|  | 26 | #23 OR #24 | 60,098 |
|  | 27 | #23 OR #25 | 60,349 |
|  | 28 | #23 OR #24 OR #25 | 60,360 |
|  | 29 | cyclophosphamide | 76,337 |
|  | 30 | "Endoxan" | 480 |
|  | 31 | #29 OR #30 | 76,397 |
|  | 32 | methotrexate | 56,506 |
|  | 33 | immunosuppressive | 565,587 |
|  | 34 | rituximab | 26,196 |
|  | 35 | "Mabthera" | 197 |
|  | 36 | #34 OR #35 | 26,208 |
|  | 37 | tocilizumab | 4,827 |
|  | 38 | "Actemra" | 67 |
|  | 39 | #37 OR #38 | 4,831 |
|  | 40 | satralizumab | 38 |
|  | 41 | "Enspryng" | 3 |
|  | 42 | #40 OR #41 | 38 |
|  | 43 | inebilizumab | 58 |
|  | 44 | "Uplizna" | 3 |
|  | 45 | #43 OR #44 | 58 |
|  | 46 | eculizumab | 2,075 |
|  | 47 | "Soliris" | 57 |
|  | 48 | #46 OR #47 | 2,077 |
|  | 49 | monoclonal antibody | 344,567 |
|  | 50 | #14 OR #20 OR #22 OR #28 OR #31 OR #32 OR #33 OR #36 OR #39 OR #42 OR #45 OR #48 OR #49 | 916,183 |
| O | 51 | relapse | 654,059 |
|  | 52 | disability | 385,924 |
|  | 53 | "Expanded Disability Status Scale" | 4,693 |
|  | 54 | EDSS | 5,023 |
|  | 55 | #52 OR #53 | 385,924 |
|  | 56 | #52 OR #54 | 386,839 |
|  | 57 | "adverse event*" | 182,044 |
|  | 58 | "adverse effect*" | 1,938,438 |
|  | 59 | "adverse reaction*" | 68,570 |
|  | 60 | "adverse drug reaction*" | 23,503 |
|  | 61 | "infusion related reaction" | 141 |
|  | 62 | #53 OR 54 OR #56 OR #57 OR #58 OR #59 OR #60 OR #61 | 2,443,598 |
| P&I | 63 | #11 AND #50 | 1,089 |
| P&I&O | 64 | #11 AND #50 AND #62 | 387 |

The search term with gray paint color box were excluded because there was no additional information found.

**eTable 1.2** SCOPUS database search result

|  | Search NO | Query | Results (title abstract keyword) |
| --- | --- | --- | --- |
| P | 1 | "neuromyelitis optica" | 5520 |
|  | 2 | NMO | 5837 |
|  | 3 | NMOSD | 1569 |
|  | 4 | "Devic's disease" | 375 |
|  | 5 | #1 OR #2 OR #3 | 9226 |
|  | 6 | #1 OR #2 OR #3 OR #4 | 9301 |
| I | 7 | azathioprine | 92105 |
|  | 8 | Imuran | 2778 |
|  | 9 | #7 OR #8 | 92179 |
|  | 10 | "mycophenolate mofetil" | 26198 |
|  | 11 | MMF | 10148 |
|  | 12 | Cellcept | 3092 |
|  | 13 | #10 OR #11 OR #12 | 33780 |
|  | 14 | "mycophenolic acid" | 45383 |
|  | 15 | Myfortic | 641 |
|  | 16 | #14 OR #15 | 45394 |
|  | 17 | #13 OR #16 | 66385 |
|  | 18 | c?closporin* | 152109 |
|  | 19 | Sandimmun* | 5841 |
|  | 20 | Neoral | 4728 |
|  | 21 | #18 OR #19 | 152122 |
|  | 22 | #18 OR #20 | 152139 |
|  | 23 | #18 OR #19 OR #20 | 152148 |
|  | 24 | cyclophosphamide | 215416 |
|  | 25 | Endoxan | 4811 |
|  | 26 | #24 OR #25 | 215614 |
|  | 27 | methotrexate | 171131 |
|  | 28 | immunosuppress* | 323822 |
|  | 29 | rituximab | 64102 |
|  | 30 | Mabthera | 2068 |
|  | 31 | #29 OR #30 | 64114 |
|  | 32 | tocilizumab | 11983 |
|  | 33 | Actemra | 580 |
|  | 34 | #32 OR #33 | 12045 |
|  | 35 | satralizumab | 87 |
|  | 36 | Enspryng | 5 |
|  | 37 | #35 OR #36 | 87 |
|  | 38 | inebilizumab | 136 |
|  | 39 | Uplizna | 4 |
|  | 40 | #38 OR #39 | 136 |
|  | 41 | eculizumab | 4233 |
|  | 42 | Soliris | 520 |
|  | 43 | #41 OR #42 | 4237 |
|  | 44 | "monoclonal antibody" | 325619 |
|  | 45 | #9 OR #17 OR #23 OR #26 OR #27 OR #28 OR #31 OR #34 OR #35 OR #38 OR #43 OR #44 | 1002833 |
| O | 46 | relapse | 201019 |
|  | 47 | disability | 409193 |
|  | 48 | "Expanded Disability Status Scale" | 9119 |
|  | 49 | EDSS | 5828 |
|  | 50 | #47 OR #48 | 409193 |
|  | 51 | #47 OR #49 | 410219 |
|  | 52 | adverse  PRE/3  ( event*  OR  effect*  OR  reaction* ) | 897683 |
|  | 53 | "infusion related reaction" | 4013 |
|  | 54 | #46 OR #51 OR #52 OR #53 | 1478514 |
| P&I | 55 | #6 AND #45 | 1849 |
| P&I&O | 56 | #6 AND #45 AND #54 | 924 |

The search term with gray paint color box were excluded because there was no additional information found.

# **Appendix 2** Risk of Bias assessment

# **eFigure 2.1** Risk of bias assessment

**
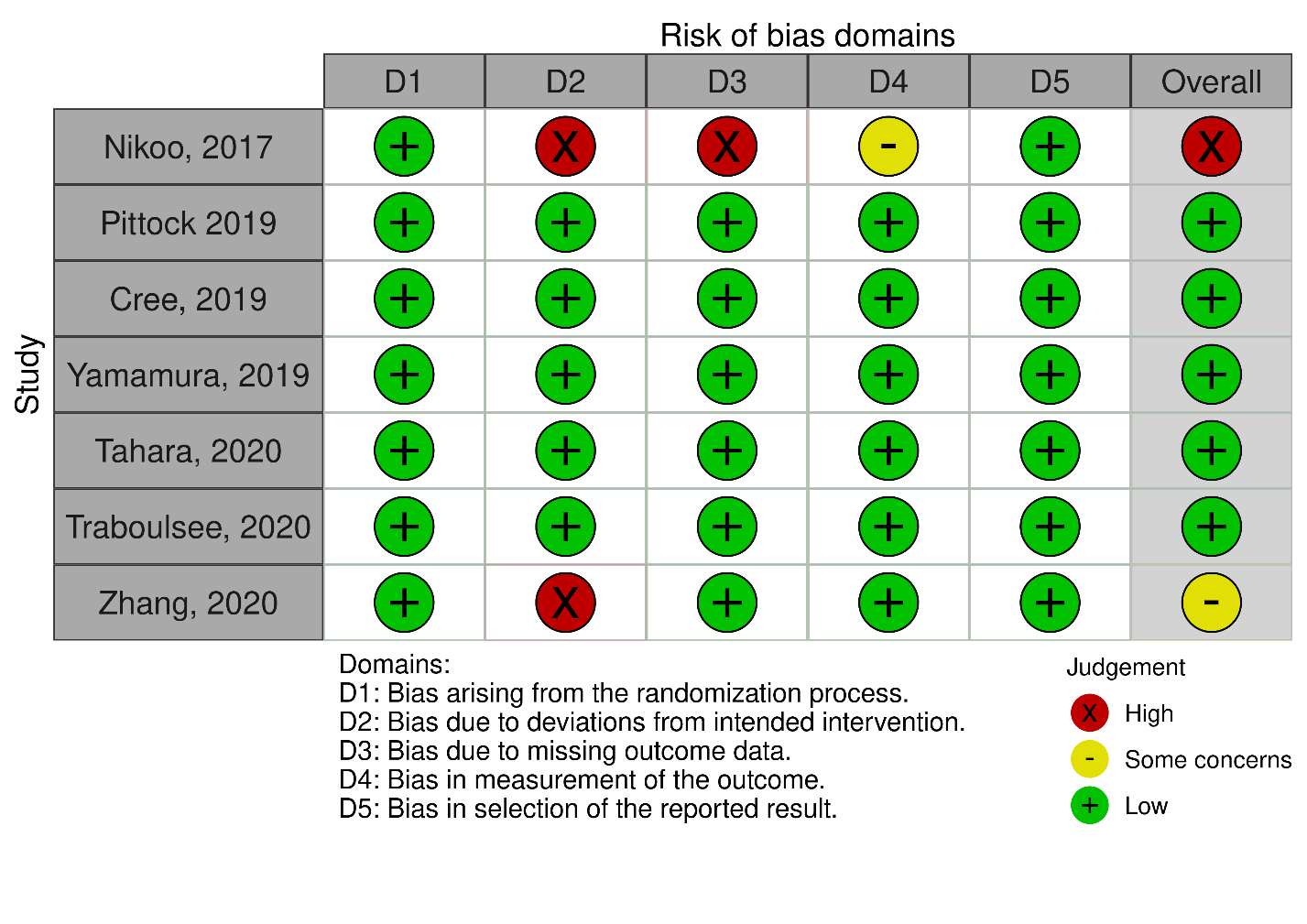
**

**Appendix 3 Results of Mixed effect parametric survival model using difference distribution**

**eTable 3.1** Mixed effect parametric survival model using difference distribution

| **Survival distribution** | **Log likelihood** | **AIC** | **BIC** |
| --- | --- | --- | --- |
| **Log-normal*** | -858.97 | 1729.95 | 1757.31 |
| **Weibull** | -863.36 | 1738.72 | 1766.09 |
| **Exponential** | -865.17 | 1740.37 | 1763.18 |
| **Gamma** | -864.00 | 1739.99 | 1767.36 |
| **Log-logistic** | -860.04 | 1732.09 | 1759.45 |

* Log-normal distribution consider as final model in the analysis of hazard ratio and median time to relapse

**Appendix 4 Funnel plots for each treatment comparison from NMA**

**eFigure 4.1** Funnel plots for each treatment comparison from NMA on time to relapse


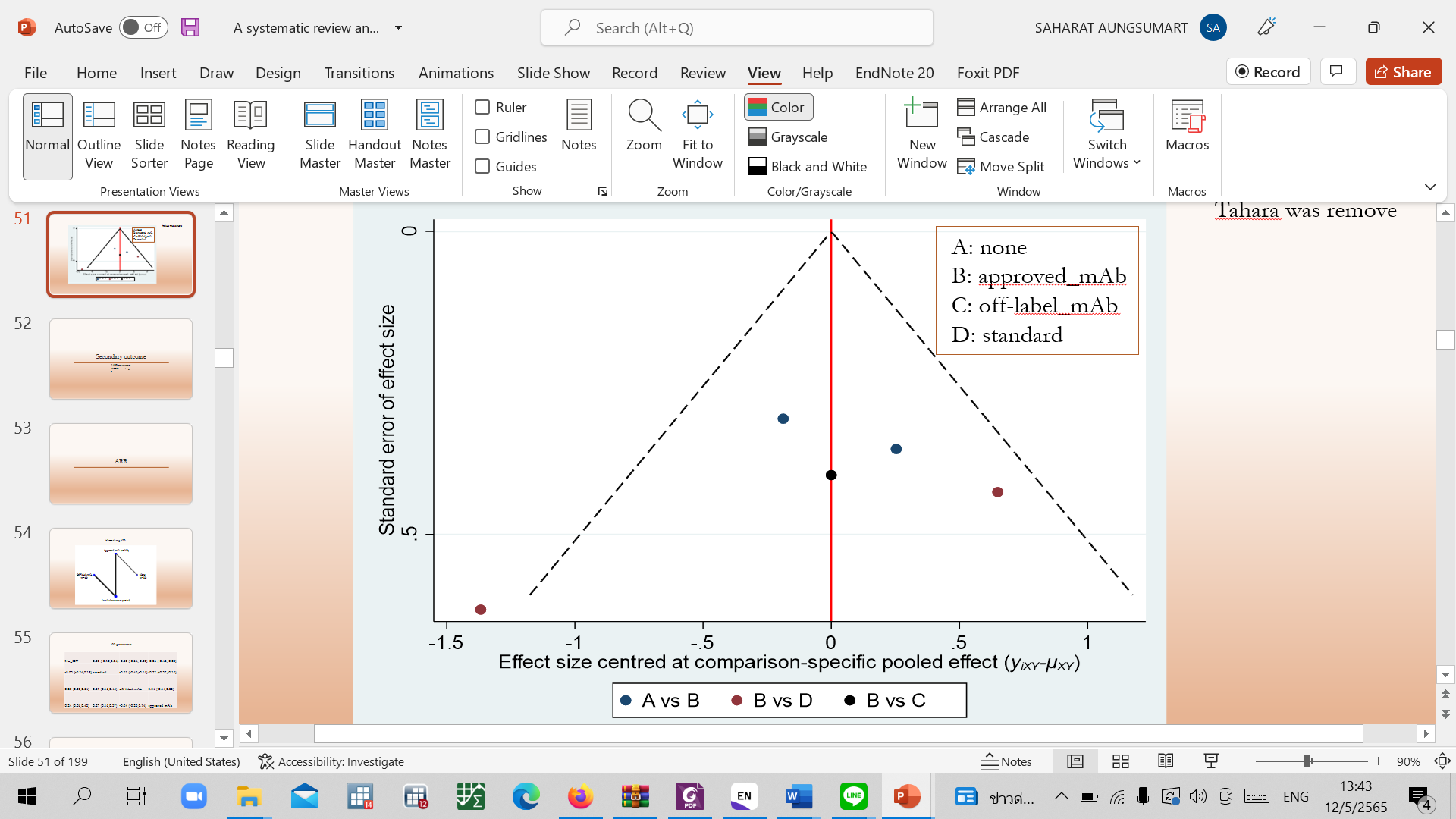


**eFigure 4.2** Funnel plots for each treatment comparison from NMA on post-treatment annualized relapse rate


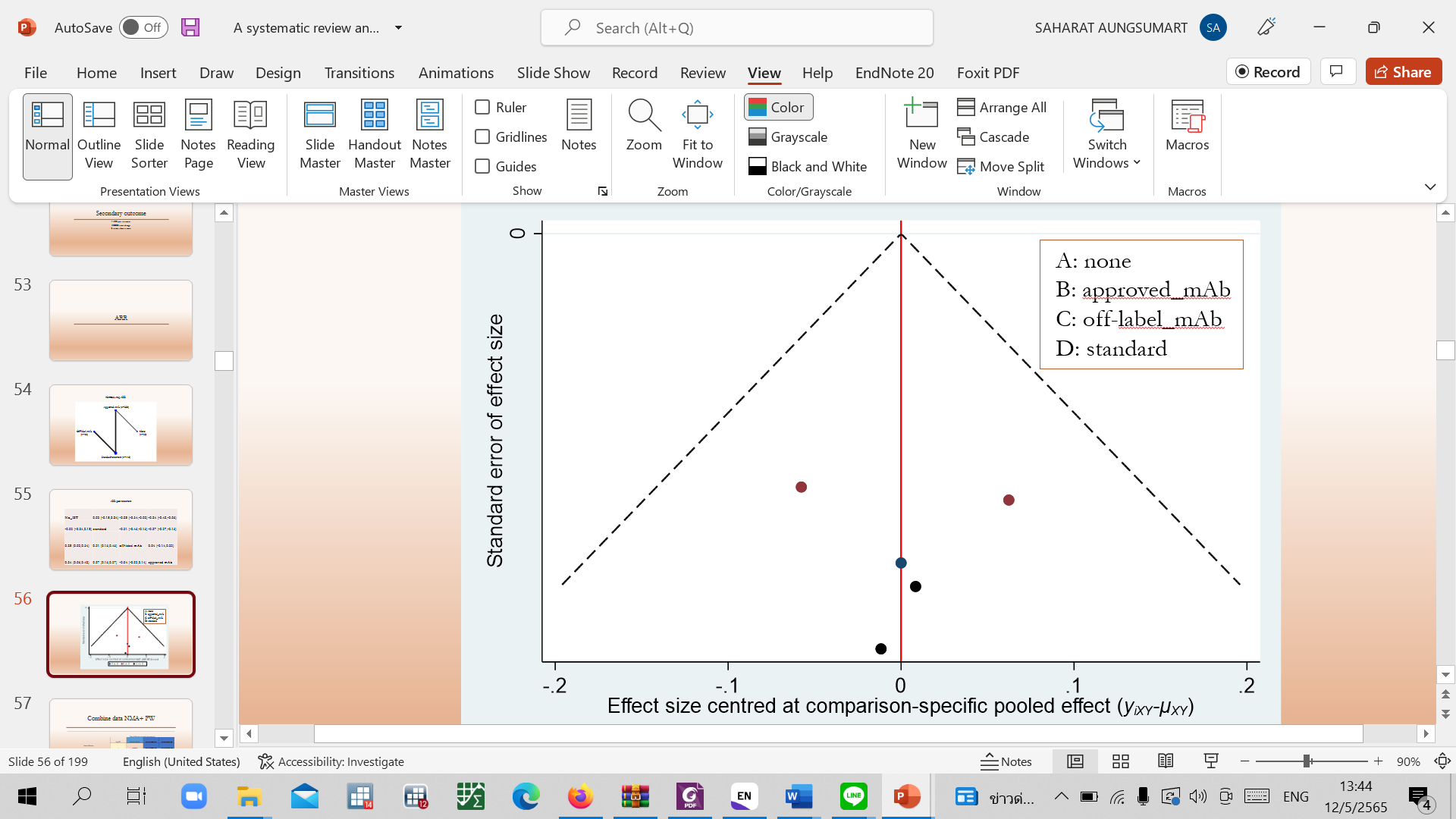


**eFigure 4.3** Funnel plots for each treatment comparison from NMA on expanded disability status score change


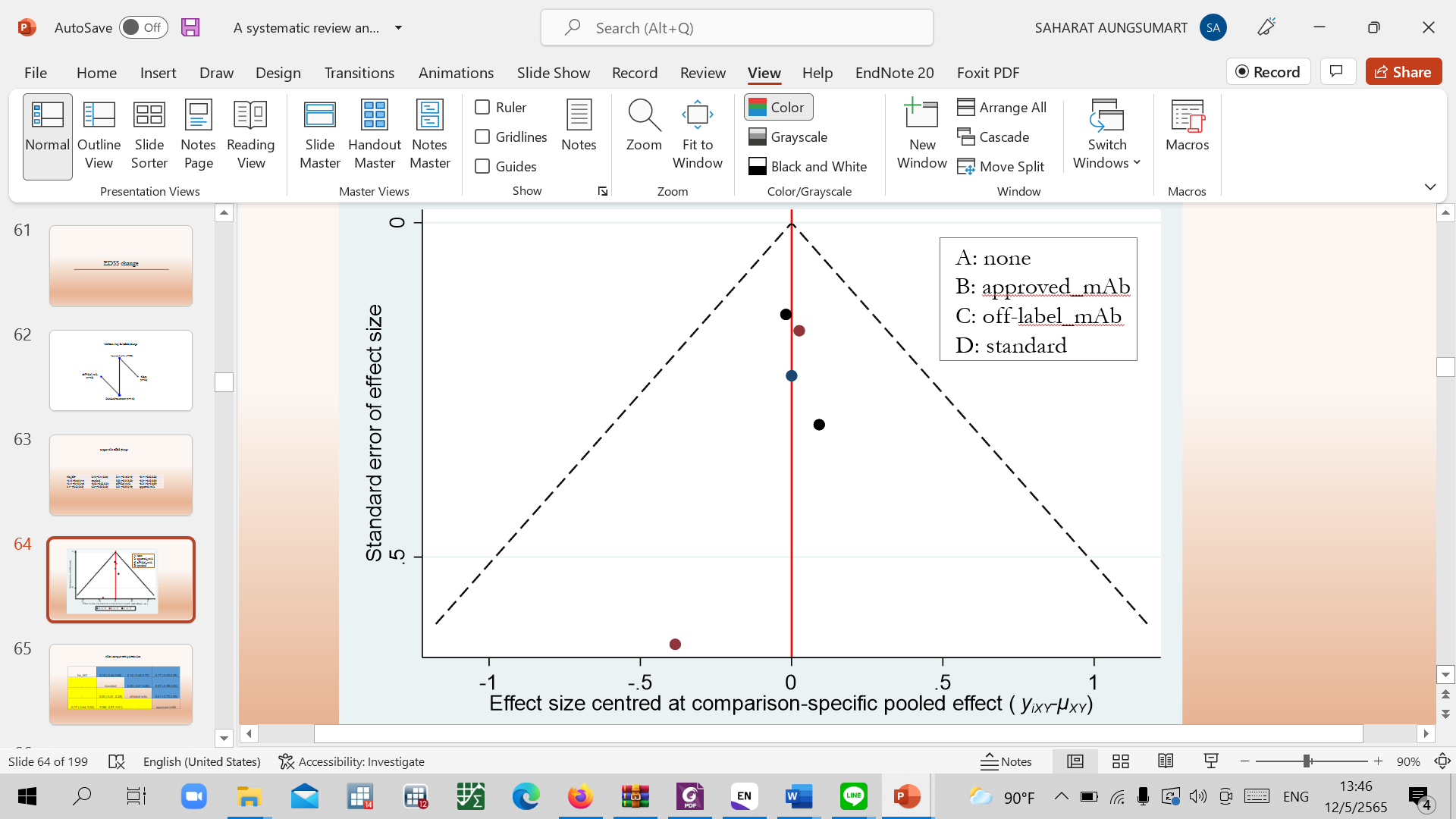


**eFigure 4.4** Funnel plots for each treatment comparison from NMA on serious adverse events


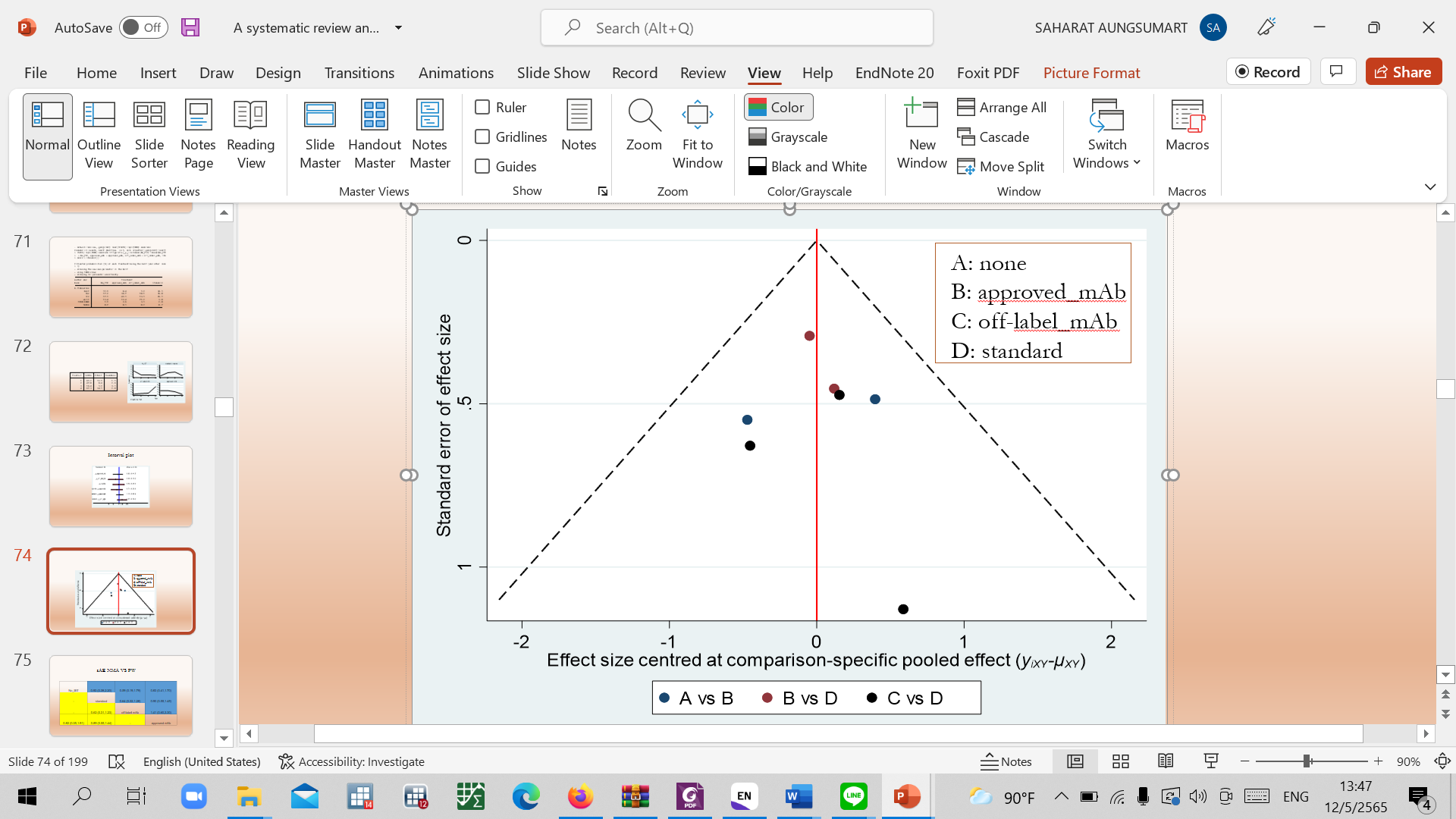


**Appendix 5** Individual study data use for analysis

**eTable 5.1** Individual study data use for analysis

| id | author | sample | treatment code | treatment group | N each arm | age (years) | % female | base line ARR | base line EDSS | AQP4 positivity | ARR post treatment | SD ARR post treatment | EDSS change | SD EDSS change | SAE |
| --- | --- | --- | --- | --- | --- | --- | --- | --- | --- | --- | --- | --- | --- | --- | --- |
| 1 | Z.Nikoo | 35 | azathioprine | standard | 35 | 32.4 | 80.0 | 1.00 | 2.40 | 57 | 0.51 | 0.55 | -0.49 | 0.59 | 3 |
|  | Z.Nikoo | 33 | rituximab | off_label_mAb | 33 | 35.3 | 87.9 | 1.30 | 3.55 | 39 | 0.21 | 0.42 | -0.44 | 0.54 | 1 |
| 2 | S.J. Pittock | 143 | eculizumab | approved_mAb | 96 | 43.9 | 92.0 | 1.94 | 4.00 | 100 | 0.02 | 0.05 | -0.18 | 0.81 | 25 |
|  | S.J. Pittock | 143 | mixed_ITS | standard | 47 | 45.0 | 89.0 | 2.07 | 4.00 | 100 | 0.35 | 0.52 | 0.12 | 0.95 | 13 |
| 3 | B. Cree | 231 | inebilizimab | approved_mAb | 175 | 43.0 | 91.0 | 1.73 | 3.81 | 92 | NA | NA | NA | NA | 8 |
|  | B. Cree | 231 | none | none | 56 | 42.6 | 89.0 | 1.57 | 4.91 | 93 | NA | NA | NA | NA | 5 |
| 4 | T.Yamamura | 83 | satralizumab | approved_mAb | 41 | 40.8 | 90.0 | 1.50 | 3.83 | 66 | 0.11 | 0.19 | -0.10 | 2.15 | 7 |
|  | T.Yamamura | 83 | mixed_ITS | standard | 42 | 43.4 | 95.0 | 1.40 | 3.63 | 67 | 0.32 | 0.42 | -0.21 | 3.46 | 9 |
| 5 | M.Tahara | 38 | rituximab | off_label_mAb | 19 | 51.5 | 90.0 | 1.70 | 3.88 | 100 | 0.00 | 0.00 | -0.32 | 0.67 | 4 |
|  | M.Tahara | 38 | corticosteroid | standard | 19 | 49.0 | 100.0 | 1.10 | 4.00 | 100 | 0.32 | 0.44 | -0.26 | 1.13 | 4 |
| 6 | A.Traboulsee | 95 | satralizumab | approved_mAb | 63 | 45.3 | 73.0 | 1.40 | 3.90 | 65 | 0.17 | 0.28 | -0.34 | 1.13 | 12 |
|  | A.Traboulsee | 95 | none | none | 32 | 40.5 | 97.0 | 1.50 | 3.70 | 72 | 0.41 | 0.49 | -0.17 | 1.01 | 5 |
| 7 | C. Zhang | 59 | tocilizumab | off_label_mAb | 59 | 48.1 | 93.0 | 1.71 | 4.50 | 85 | NA | NA | NA | NA | 6 |
|  | C. Zhang | 59 | azathioprine | standard | 59 | 45.3 | 90.0 | 1.68 | 4.50 | 90 | NA | NA | NA | NA | 11 |

Abbreviations: AQP4, Aquaporin4; ARR, Annualized relapse rate; EDSS, expanded disability status score; mAb, monoclonal antibody; NA, not available; SAEs, serious adverse events; SD, standard deviation.
